# Extracellular Vesicle Proteins and MicroRNAs Are Linked to Chronic Post-Traumatic Stress Disorder Symptoms in Service Members and Veterans With Mild Traumatic Brain Injury

**DOI:** 10.3389/fphar.2021.745348

**Published:** 2021-10-06

**Authors:** Vivian A. Guedes, Chen Lai, Christina Devoto, Katie A. Edwards, Sara Mithani, Dilorom Sass, Rany Vorn, Bao-Xi Qu, Heather L. Rusch, Carina A. Martin, William C. Walker, Elisabeth A. Wilde, Ramon Diaz-Arrastia, Jessica M. Gill, Kimbra Kenney

**Affiliations:** ^1^ Tissue Injury Branch, National Institutes of Nursing Research, National Institutes of Health, Bethesda, MD, United States; ^2^ Center for Neuroscience and Regenerative Medicine, Uniformed Services University, Bethesda, MD, United States; ^3^ Center for Neuroscience and Rehabilitation Medicine, Uniformed Services University of the Health Sciences and National Institutes of Health, Bethesda, MD, United States; ^4^ Henry M. Jackson Foundation for the Advancement of Military Medicine, Bethesda, MD, United States; ^5^ Department of Physical Medicine and Rehabilitation, Virginia Commonwealth University, Richmond, VA, United States; ^6^ CENC Neuroimaging Core, George E. Wahlen VA Salt Lake City Healthcare System and Traumatic Brain Injury and Concussion Center, Department of Neurology, University of Utah, Salt Lake City, UT, United States; ^7^ Department of Neurology, University of Pennsylvania Perelman School of Medicine, Philadelphia, PA, United States; ^8^ CENC Biorepository, Uniformed Services University of the Health Sciences, Bethesda, MD, United States; ^9^ Johns Hopkins University School of Nursing and Medicine, Baltimore, MD, United States; ^10^ Department of Neurology, Uniformed Services University of the Health Sciences, Bethesda, MD, United States

**Keywords:** concussion, biomarkers, exosomes, military, PTSD, neurofilament light (NfL)

## Abstract

Symptoms of post-traumatic stress disorder (PTSD) are common in military populations, and frequently associated with a history of combat-related mild traumatic brain injury (mTBI). In this study, we examined relationships between severity of PTSD symptoms and levels of extracellular vesicle (EV) proteins and miRNAs measured in the peripheral blood in a cohort of military service members and Veterans (SMs/Vs) with chronic mTBI(s). Participants (*n* = 144) were divided into groups according to mTBI history and severity of PTSD symptoms on the PTSD Checklist for DSM-5 (PCL-5). We analyzed EV levels of 798 miRNAs (miRNAs) as well as EV and plasma levels of neurofilament light chain (NfL), Tau, Amyloid beta (Aβ) 42, Aβ40, interleukin (IL)-10, IL-6, tumor necrosis factor-alpha (TNFα), and vascular endothelial growth factor (VEGF). We observed that EV levels of neurofilament light chain (NfL) were elevated in participants with more severe PTSD symptoms (PCL-5 ≥ 38) and positive mTBI history, when compared to TBI negative controls (*p* = 0.024) and mTBI participants with less severe PTSD symptoms (*p* = 0.006). Levels of EV NfL, plasma NfL, and hsa-miR-139–5p were linked to PCL-5 scores in regression models. Our results suggest that levels of NfL, a marker of axonal damage, are associated with PTSD symptom severity in participants with remote mTBI. Specific miRNAs previously linked to neurodegenerative and inflammatory processes, and glucocorticoid receptor signaling pathways, among others, were also associated with the severity of PTSD symptoms. Our findings provide insights into possible signaling pathways linked to the development of persistent PTSD symptoms after TBI and biological mechanisms underlying susceptibility to PTSD.

## Introduction

Post-traumatic stress disorder (PTSD) is highly prevalent in military populations and frequently associated with deployment-related mild traumatic brain injury (mTBI) (Hoge et al., 2008; Schneiderman et al., 2008). The risk for the development of PTSD after exposure to traumatic experiences varies among individuals and populations (Holmes and Singewald, 2013), and biological mechanisms underlying susceptibility to PTSD development remain largely unknown. Finding biomarkers associated with persistent PTSD symptoms following mTBIs may shed light on the underlying pathobiology and may lead to novel molecular targets for the development of personalized therapies.

Extracellular vesicles (EVs) such as exosomes and microvesicles are released by cells throughout the body, including neurons and glia (Fauré et al., 2006; Krämer-Albers et al., 2007). EVs have a lipid bilayer and contain a cargo that includes proteins (e.g., cytokines and growth factors) and microRNAs (miRNAs) (Taylor and Gercel-Taylor 2013; Snijders et al., 2018), which are small non-coding RNAs that are thought to play a major role in the regulation of gene expression and epigenetic alterations (Taylor and Gercel-Taylor, 2013). EVs have been linked to the pathology of age-related neurodegenerative conditions, play a role in angiogenesis and have immunodulatory properties (Perez-Gonzalez et al., 2012; Dinkins et al., 2017; DeLeo and Ikezu, 2018; Oggero et al., 2019). Moreover, neuroinflammatory activity can be initiated by EVs, which contain biologically active cytokines in their cargo and surface (Gupta and Pulliam, 2014; Fitzgerald et al., 2018). Importantly, EVs readily cross the blood brain barrier (BBB) to the peripheral circulation, where they can be accessed and isolated, allowing for the quantification of their protein and miRNA content (Gupta and Pulliam, 2014; Fitzgerald et al., 2018).

TBI of all severities has been associated with remote neurodegeneration, persistent inflammation, and vascular changes (Johnson et al., 2013; Woodcock and Morganti-Kossmann, 2013; Mckee and Daneshvar, 2015; Jassam et al., 2017). PTSD has been linked to inflammation and immune dysregulation, with reports of elevated peripheral blood levels of cytokines in association with PTSD symptoms in participants with TBI (Gill et al., 2010; Passos et al., 2015; O’Donovan et al., 2017; Rodney et al., 2020). Our group has previously reported correlations between PTSD symptom severity and EV levels of neurofilament light chain (NfL), a protein found in large myelinated axons and a marker of axonal injury and degeneration in military populations with a positive history of mTBI (Menke et al., 2015; Zetterberg 2016; Guedes et al., 2020a). Differential expression of circulating miRNAs has been observed in mTBI as well as PTSD (Bhomia et al., 2016; Gheysarzadeh et al., 2018; Lee et al., 2019; Snijders et al., 2019). Nevertheless, the potential of EV proteins and miRNAs as biomarkers of PTSD and/or PTSD symptoms in chronic TBI populations remains largely unexplored.

In this study, we aimed to analyze the molecular signature associated with severity of persistent PTSD symptoms in a cohort of combat-exposed service members and Veterans (SMs/Vs) with and without remote mTBI(s). The levels of 798 miRNAs in peripherally circulating EVs were evaluated. In addition, we measured the EV and plasma levels of eight proteins: NfL, Tau, Amyloid beta (Aβ) 42, Aβ40, interleukin-6 (IL-6), IL-10, tumor-necrosis factor-alpha (TNFα), as well as vascular endothelial growth factor (VEGF). NfL, tau, Aβ42, and Aβ40 are candidate TBI biomarkers that have been linked to neurodegenerative processes (Brody et al., 2015; Mustapic et al., 2017; Khalil et al., 2018); whereas IL-10, IL-6, TNFα are cytokines implicated in inflammatory responses (Morganti-Kossmann et al., 2002; Taylor and Gercel-Taylor 2014); and VEGF is an angiogenesis and vascular injury marker (Arany et al., 2008; Vempati et al., 2014).

## Materials and Methods

### Study Design and Population

Participants in the current study were all enrolled in the Chronic Effects of Neurotrauma Consortium (CENC) Multicenter Prospective Longitudinal Study (PLS), an observational study of mTBI among post-9/11 era SMs/Vs (Walker et al., 2018), now funded under the Long Term Impact of Military Brain Injury Consortium (LIMBIC). The LIMBIC-CENC PLS study assesses SMs/Vs for all potential concussive events (PCEs) during lifetime and determines a mTBI diagnosis for each event based on DoD-VA common definition of mTBI. Blood was stored in the CENC biorepository until analyzed. The inclusion criteria for the parent study were: 1) a history of Operation Enduring Freedom (OEF)/Operation Iraqi Freedom (OIF)/Operation New Dawn (OND) deployment confirmed by Veterans Affairs (VA) or the United States Department of Defense (DoD) records; 2) a history of combat exposure during any deployment; 3) adult age >18 years. Exclusion criteria included the following: 1) history of moderate, severe or penetrating TBI (i.e., initial Glasgow Coma Scale <13, coma duration >½ hrs., post-traumatic amnesia duration >24 h, or traumatic intracranial lesion on head CT); 2) history of 1) major neurologic disorder (e.g., stroke, spinal cord injury), 2) major psychiatric disorder (e.g., schizophrenia) defined as resulting in a significant decrement in functional status or loss of independent living capacity. Common neurologic (e.g., mini-stroke, neuropathy) and common psychiatric comorbidities (e.g., depression, bipolar disorder) were permitted.

The sample for the current study, a cross-sectional analysis of baseline evaluations, was derived from an early ‘Snapshot’ Biomarker Discovery dataset of participants enrolled at the four original CENC sites (Veteran Affairs Medical Centers: in Richmond, VA; Tampa, FL; Houston, TX; San Antonio, TX). All participants provided written informed consent to participate, and all sites received approval from their respective institutional review boards, including blood collection, and followed their ethical standards. A convenience sample of 151 participants with available EV protein and miRNA data was analyzed in this study. Participants were classified into four groups based on TBI history and severity of PTSD symptoms as measured by the PTSD Checklist for DSM-5 (PCL-5) (Blevins et al., 2015): 1) Control, subjects (i.e. -TBI and -PTSD) negative for any lifetime TBI and PCL-5 score under 38 (*n* = 28); 2) +mTBI/-PTSD, participants with positive history of mTBI and with PCL-5 score under 38 (*n* = 71); 3) +mTBI/+ PTSD, mTBI participants with PCL-5 score of 38 or higher (*n* = 45). Participants without prior mTBI and with PCL-5 score equal or higher than 38 (*n* = 7) were excluded from the analysis, and the remaining 144 participants were included in this study. Investigators were blinded to the group allocation for protein and miRNA analysis.

### Assessment of Post-Traumatic Stress Disorder Symptoms

PCL-5 is a 20-item questionnaire, corresponding to the DSM-5 symptom criteria for PTSD. PCL-5 results in an overall score of 0–80, obtained by summing the individual scores (range 0–4) for the 20 items. Higher PCL-5 scores indicate a greater symptom burden. PCL-5 is commonly used to assess PTSD symptoms and to help determine appropriate steps and treatment options for patients who may have PTSD (Blevins et al., 2015; Bovin et al., 2016). The PCL-5 cutoff of 38 is comparable to the established and validated 17-item PCL cutoff of 50 in military populations (Hoge et al., 2014).

### Determination of Traumatic Brain Injury

Using a modification of the Ohio State University TBI Identification (OSU TBI-ID) instrument (Corrigan and Bogner, 2007), participants were screened for PCEs sustained during military deployments and across their entire lifetime, including childhood. Each PCE was further investigated via the Virginia Commonwealth University Retrospective Concussion Diagnostic Interview (VCU rCDI) (Walker et al., 2015), which rendered a preliminary mTBI diagnosis for each event based on DoD-VA common definition of mTBI. The preliminary TBI diagnosis was reviewed by the site principal investigator, and vetted against the unstructured free text portion of the interview and available medical documents recorded in proximity to the event (i.e., first responder, emergency department, or in-theatre documentation) as previously described (Walker et al., 2018). The TBI severity of each event was also evaluated and if any event was assessed more severe than an mTBI, the participant was excluded from the study. Additionally, if any uncertainty regarding the TBI diagnosis remained, the event was adjudicated by a central diagnosis committee consisting of national experts in TBI. Using these TBI determinations, participants were classified into mTBI positive or TBI negative control group and the lifetime number of mTBIs per individual was summed.

### Laboratory Methods

#### Extraction of Extracellular Vesicle miRNA

Whole blood samples were collected and, within 2 h, were centrifuged at 3,000 rpm for 10 min (4°C). Plasma aliquots were stored at −80°C until analyzed. Plasma samples were thawed on ice and centrifuged at 3,000 rpm for 5 min to pellet and removal of cells and debris. Qiagen ExoRNeasy Serum/Plasma Kits (Qiagen, Germantown, MD, United States) was used to extract miRNAs from EVs as per manufacturer’s instructions. For miRNA extraction, 400 µL of plasma samples per participant was used, and miRNAs were eluted in 20 µL of water. After this, the RNA extract was cleaned and concentrated using the RNA Clean and Concentrator-5 kit (Zymo Research Corp., Irvine, CA, United States), and eluted in 10 μL of water. The concentration, purity, and integrity of the EV miRNA product was determined using Bioanalyzer (Agilent, Santa Clara, CA, United States) and Qubit (Thermo Fisher Scientific, Waltham, MA, United States).

#### miRNA Profiling Analysis

Analysis was performed with nCounter® Human v3 miRNA Expression Panels (NanoString Technologies, WA, United States). The expression panel contained 798 miRNA probes; this was the maximum number of probes available for analysis in human samples. Spike-in synthetic targets were introduced to control for variability in miRNA extraction and ligation: Arabidopsis thaliana miR-159a (ath-miR-159a), Caenorhabditis elegans (cel)-miR-248 and miR-254, and Oryza sativa (osa)-miR-414 and 442. They are incorporated in the code sets and used for analysis along with positive and negative controls. All hybridizations took place around 18 h, and all counts were obtained at MAX mode, with the creation of 555 images per sample. Analysis of raw miRNA data was performed using the nSolver analysis software (version 4.0, NanoString technologies). Code count normalization was performed with the geometric median for the 50 highest expressed genes. MiRNAs with an *p*-value of less than 0.05 (after adjustment for false discovery rate, FDR) were considered as differentially expressed and used for subsequent analysis. Target filter analysis (Ingenuity Systems Inc., CA, United States), which allows the identification of biologically relevant targets, was performed for relevant miRNAs. In the analysis, we prioritized human studies with experimentally validated and predicted mRNA targets, with targets related to inflammatory responses, neurological disease, cardiovascular disease, organismal injury and abnormalities and psychological disorders.

#### Extracellular Vesicle Isolation for Protein Analysis

We used 500 µL of frozen human plasma to obtain EVs for protein analysis. EVs were precipitated by using ExoQuick™ Plasma Prep and Exosome Precipitation Kit (EXOQ5TM-1, System Biosciences Inc., Mountainview, CA, United States), which allow EV isolation at low gravitational centrifugal forces (Peterson et al., 2015). Plasma samples were first treated with thrombin and incubated at room temperature for 5–10 min. Then, samples were centrifuged at 10,000 rpm for 5 minutes and the supernatant was transferred into a clean tube for EV isolation. After this, we added 126 µL of ExoQuick solution to each thrombin-treated plasma sample, mixing well by inverting the tube, and incubated the resulting mixture for 30 min at 4°C. Tubes were kept upright during incubation. Then, vials were centrifuged at 1,500 × g for 30 min, according to the manufacturer’s instructions. After the centrifugation, EVs appeared as pellets at the bottom of the tube. The supernatant was aspirated from each tube, and each pellet was resuspended in 500 µL of 1 x phosphate-buffered saline (PBS). Samples were then stored at −80°C. We used TSG101 (EV and exosome marker) ELISA to confirm the presence of EVs in the samples.

#### Protein Quantification

For the protein quantification, each sample received equal amounts of mammalian protein extraction reagent (M-PER) to lyse EVs (Thermo Scientific, Inc., Rockford, IL, United States), containing three times the suggested concentrations of protease inhibitors (cOmplete™ ULTRA Tablets protease Inhibitor Cocktail, MiliporeSigma, Burlington, MA, United States). These suspensions were used to measure protein concentrations. EV and plasma levels of NfL (NF-light Simoa Assay, item 103,186, Quanterix, Lexington, MA, United States), Tau, Aβ42, Aβ40 (Neurology 3-Plex A, item 101,995, Quanterix), IL-10, IL-6, TNFα (Cytokine 3-Plex A, item 101,160, Quanterix), and VEGF (VEGF Discovery Kit, item 102,794, Quanterix) were analyzed using a Simoa HD-1 analyzer (Quanterix), according to the manufacturer’s instructions. The Simoa HD-1 analyzer uses an ultrasensitive paramagnetic bead-based enzyme-linked immunosorbent assay. Each sample was analyzed in duplicates. Samples with coefficients of variance (CVs) higher than 20% were excluded from subsequent analysis.

### Statistical Analysis

Comparison of demographic, and clinical characteristics between groups were conducted using Chi-square test (χ^2^), Mann-Whitney *U* tests, and Kruskal-Wallis test. Non-parametric tests were used for biomarker analysis, instead of parametric tests, as data were not normally distributed. Comparisons of miRNA levels between groups were performed by using Kruskal-Wallis test followed by Dunn’s test and Benjamini-Hochberg for FDR. miRNAs with adjusted *p* < 0.05 were considered differentially expressed. For the protein analysis, we used Kruskal-Wallis test followed by Dunn’s test, and Bonferroni method to correct for multiple comparisons. We examined relationships between PTSD symptoms and differentially expressed miRNA and protein levels by using Spearman correlations tests as well as linear regression models. Linear regression models were built for each of the statistically significant biomarkers, and included PCL-5 scores as the outcome, controlling for potential confounders (demographics, total number of mTBIs and time since the last mTBI). All data were analyzed using R (version 4.0.2) statistical packages. R and GraphPad Prism were used to produce graphs (version 8.4.3).

## Results

### Demographics and Clinical Characteristics

Demographic and clinical characteristics including PCL-5 scores of the 144 final participants are described in Table 1. The cohort was predominately male (89%) with a median age of 37 (IQR = 31–49). No significant differences on the demographic variables of age, gender, and education between mTBI/PTSD groups were observed. For TBI positive groups, TBI characteristics including total number of mTBIs, time since first mTBI and time since last mTBI were also not significantly different.

**TABLE 1:** Demographic and clinical characteristics.

Characteristic	Control (-TBI/-PTSD) (*n* = 28)	+mTBI/-PTSD (*n* = 71)	+mTBI/+PTSD (*n* = 45)	Significance
Sex (male)	25 (89%)	60 (85%)	40 (89%)	*p* = 0.723
				x^2^ = 0.648
				df = 2
Age	37 (31, 51)	38 (31, 47)	36 (30, 49)	*p* = 0.864
Education				*p* = 0.269 x^2^ = 0.65 df = 5.19
College 1 year to 3 years	10 (36%)	34 (48%)	17 (38%)	
College 4 years or more	13 (46%)	32 (45%)	19 (42%)	
High school graduate	5 (18%)	5 (7.0%)	9 (20%)	
Number of mTBIs	N/A	2.00 (1.00, 3.00)	2.00 (1.00, 3.00)	*p* = 0.837^a^
Time since first mTBI (years)	N/A	19 (12, 25)	13 (9, 26)	*p* = 0.190
Time since last mTBI (years)	N/A	9 (5, 12)	8 (5, 11)	*p* = 0.408
PCL-5 (score)	7 (3, 16)	19 (9, 28)	49 (44, 61)	N/A
PHQ9 (score)	2.0 (0.8, 4.0)	7.0 (3.0, 9.8)	14.0 (11.0, 18.0)	**p < 0.001**

Participants were classified into three groups based on TBI history and severity of PTSD symptoms as measured by PCL-5: 1) Control (i.e., -TBI and -PTSD); 2) +mTBI/-PTSD (i.e., history of TBI and PCL-5 < 38); 3) +mTBI/+PTSD (i.e., history of TBI and PCL-5 > 38). Abbreviations: TBI (traumatic brain injury); PCL-5 (PTSD Checklist for DSM-5); PHQ-9 (Patient Health Questionnaire, Version 9). Statistics presented: n (%); Median (IQR). Statistical tests performed: Chi-square test (χ2), Mann-Whitney U tests, and Kruskal-Wallis test. ^a^Compared between +mTBI/-PTSD and +mTBI/+PTSD groups. Statistically significant p values are marked in bold.

### MiRNA Analysis

We analyzed 798 miRNAs and found 12 differentially expressed miRNAs (Table 2, Supplementary Table S1). Pairwise comparisons were performed to assess differences in miRNA expression levels between groups. After correcting for FDR, differentially regulated miRNAs were as follows: +mTBI/-PTSD vs control (hsa-miR-139–5p, hsa-miR-204–5p, hsa-miR-372–3p, hsa-miR-509-3-5p, hsa-miR-615–5p, hsa-miR-1277–3p); +mTBI/+PTSD vs control (hsa-miR-3190–3p, hsa-miR-615–5p, hsa-miR-1185-1-3p, hsa-miR-3196, hsa-miR-372–3p, hsa-miR-139–5p); +mTBI/+PTSD vs +mTBI/-PTSD (hsa-miR-374a-3p).

**TABLE 2:** Differentially regulated miRNAs in group comparisons.

Pairwise comparison	miRNA	FC	Log2FC	Adj *p* ^a^	Up/down
+mTBI/+PTSD vs Control	hsa-miR-3190–3p	1.305	0.384	**0.012**	Up
	hsa-miR-615–5p	1.326	0.407	**0.033**	Up
	hsa-miR-1185-1-3p	1.191	0.252	**0.033**	Up
	hsa-miR-3196	1.368	0.452	**0.036**	Up
	hsa-miR-372–3p	1.273	0.349	**0.038**	Up
	hsa-miR-139–5p	0.867	−0.206	**0.048**	Down
	hsa-miR-375	1.300	0.378	0.059	Up
	hsa-miR-204–5p	1.188	0.248	0.087	Up
	hsa-miR-1277–3p	1.184	0.244	0.154	Up
	hsa-miR-509-3–5p	1.149	0.201	0.210	Up
	hsa-miR-425–5p	1.078	0.109	0.615	Up
	hsa-miR-374a-3p	0.994	−0.009	0.781	Down
+mTBI/+PTSD vs + mTBI/-PTSD	hsa-miR-374a-3p	0.860	−0.217	**0.041**	Down
	hsa-miR-1185-1-3p	1.131	0.178	0.057	Up
	hsa-miR-425–5p	0.886	−0.174	0.089	Down
	hsa-miR-139–5p	1.154	0.206	0.095	Up
	hsa-miR-3190–3p	1.089	0.123	0.131	Up
	hsa-miR-509-3-5p	0.918	−0.124	0.253	Down
	hsa-miR-3196	1.069	0.096	0.358	Up
	hsa-miR-204–5p	0.917	−0.125	0.374	Down
	hsa-miR-1277–3p	0.934	−0.098	0.392	Down
	hsa-miR-375	0.966	−0.049	0.868	Down
	hsa-miR-372–3p	1.004	0.005	0.892	Up
	hsa-miR-615–5p	1.031	0.044	0.973	Up
+mTBI/-PTSD vs Control	hsa-miR-139–5p	0.751	−0.413	**0.001**	Down
	hsa-miR-204–5p	1.295	0.373	**0.015**	Up
	hsa-miR-372–3p	1.269	0.344	**0.034**	Up
	hsa-miR-509-3-5p	1.252	0.324	**0.035**	Up
	hsa-miR-615–5p	1.287	0.364	**0.037**	Up
	hsa-miR-1277–3p	1.268	0.342	**0.038**	Up
	hsa-miR-375	1.345	0.428	0.054	Up
	hsa-miR-3196	1.279	0.356	0.081	Up
	hsa-miR-425–5p	1.217	0.283	0.094	Up
	hsa-miR-3190–3p	1.198	0.261	0.104	Up
	hsa-miR-374a-3p	1.155	0.208	0.107	Up
	hsa-miR-1185-1-3p	1.052	0.074	0.640	Up

a *p* values for pairwise group comparison after FDR adjustment. Statistically significant p values are marked in bold.

Participants were classified into three groups based on TBI history and severity of PTSD symptoms as measured by the PTSD Checklist for DSM-5 (PCL-5): 1) Control (i.e., -TBI and -PTSD); 2) +mTBI/-PTSD; 3) +mTBI/+PTSD. Comparisons of miRNA levels between groups were performed by using Kruskal-Wallis test followed by Dunn’s test. Benjamini-Hochberg Procedure for false discovery rate (FDR) was used to adjusted *p* values. miRNAs with adjusted *p* < 0.05 were considered differentially regulated and are marked in bold. Abbreviations: mTBI (mild traumatic brain injury), PTSD (Post-traumatic stress disorder), FC (fold change), Up (upregulated), Down (Downregulated), Adj *p* (adjusted *p* value).

**FIGURE 1:**
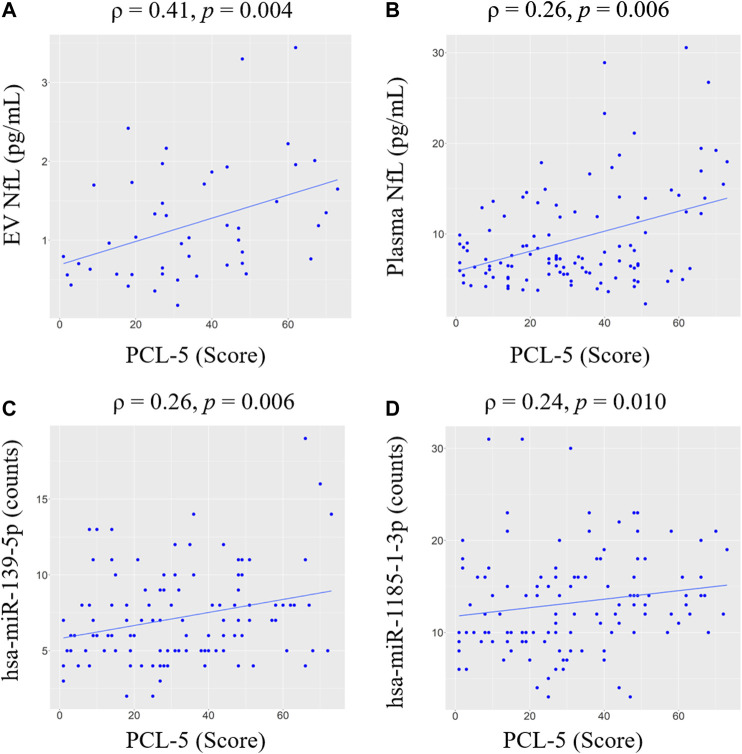
Correlations between PCL-5 scores and biomarker levels within the TBI group. Spearman correlation coefficients (ρ) and significance levels (*p*) are shown for **(A)** EV NfL, **(B)** plasma NfL, **(C)** hsa-miR-139–5p, **(D)** hsa-miR-1185-1-3p. Only significant correlations are shown. Regression line is shown in blue. Abbreviations: neurofilament light chain (NfL); PTSD Checklist for DSM-5 (PCL-5); extracellular vesicle (EV).

To evaluate relationships between miRNA levels and severity of PTSD symptoms, we also calculated correlations between levels of differentially expressed miRNA and PCL-5 scores (Figure 1, Supplementary Table S2). We observed that levels of hsa-miR-139–5p (*ρ* = 0.26, *p* = 0.006) and hsa-miR-1185-1-3p (*ρ* = 0.24, *p* = 0.010) significantly correlated with PCL-5 scores. Marginally significant correlations were observed between hsa-miR-3190–3p (*ρ* = 0.16, *p* = 0.086), hsa-miR-422–5p (*ρ* = -0.17, *p* = 0.065) and PCL-5 scores. We also built linear regression models including total number of TBIs and time since last TBI to evaluate links between PCL-5 scores and miRNA levels (Table 3). We found a significant association between PCL-5 scores and hsa-miR-139–5p. In addition, combining hsa-miR-139–5 and protein biomarkers resulted in model improvements (Table 3).

**TABLE 3:** Linear Regression Models for PCL-5 scores.

*Predictors*	*Est*	*p*	*Est*	*p*	*Est*	*p*	*Est*	*p*	*Est*	*p*
(Intercept)	54.50	**<0.001**	27.18	**0.001**	12.35	0.216	33.57	**0.012**	15.07	0.104
Age	−0.62	**0.024**	-0.43	**0.016**	0.06	0.768	−0.50	0.057	−0.27	0.192
EV NfL	12.93	**0.001**					10.36	**0.007**		
Plasma NfL			1.79	**<0.001**					1.61	**<0.001**
hsa-miR-139–5p					1.82	**0.003**	2.82	**0.002**	1.35	**0.020**
*R* ^2^/*R* ^2^ adjusted	0.287/0.221		0.216/0.186		0.094/0.052		0.460/0.381		0.263/0.221	

Linear regression models were used to evaluate links between PCL-5 Scores and biomarker levels, controlling for demographics, time since last TBI (years) and number of TBIs. Only significant predictors are shown. Models combining either EV NfL or plasma NfL and hsa-miR-139–5p are also provided. Abbreviations: Est. (estimates); traumatic brain injury (TBI); PCL-5 (PTSD Checklist for DSM-5); Neurofilament light (NfL).

To explore functional associations of differentially regulated miRNAs linked to the development of more severe TBI symptoms, we performed a target filter analysis including miRNAS that were differentially expressed in the +mTBI/+PTSD group when compared to the +mTBI/-PTSD and control groups. The target analysis revealed canonical pathways linked to nervous system physiology, neurodegenerative diseases, mitochondrial dysfunction, oxidative phosphorylation, immunological function and inflammatory responses (e.g, IL-6 and IL-10), VEGF signaling, cardiac hypertrophy and cardiac beta-adrenergic signaling, as well as insulin secretion, estrogen receptor, and glucocorticoid receptor signaling canonical pathways (Supplementary Figures S1–S4).

### Protein Analysis

**FIGURE 2:**
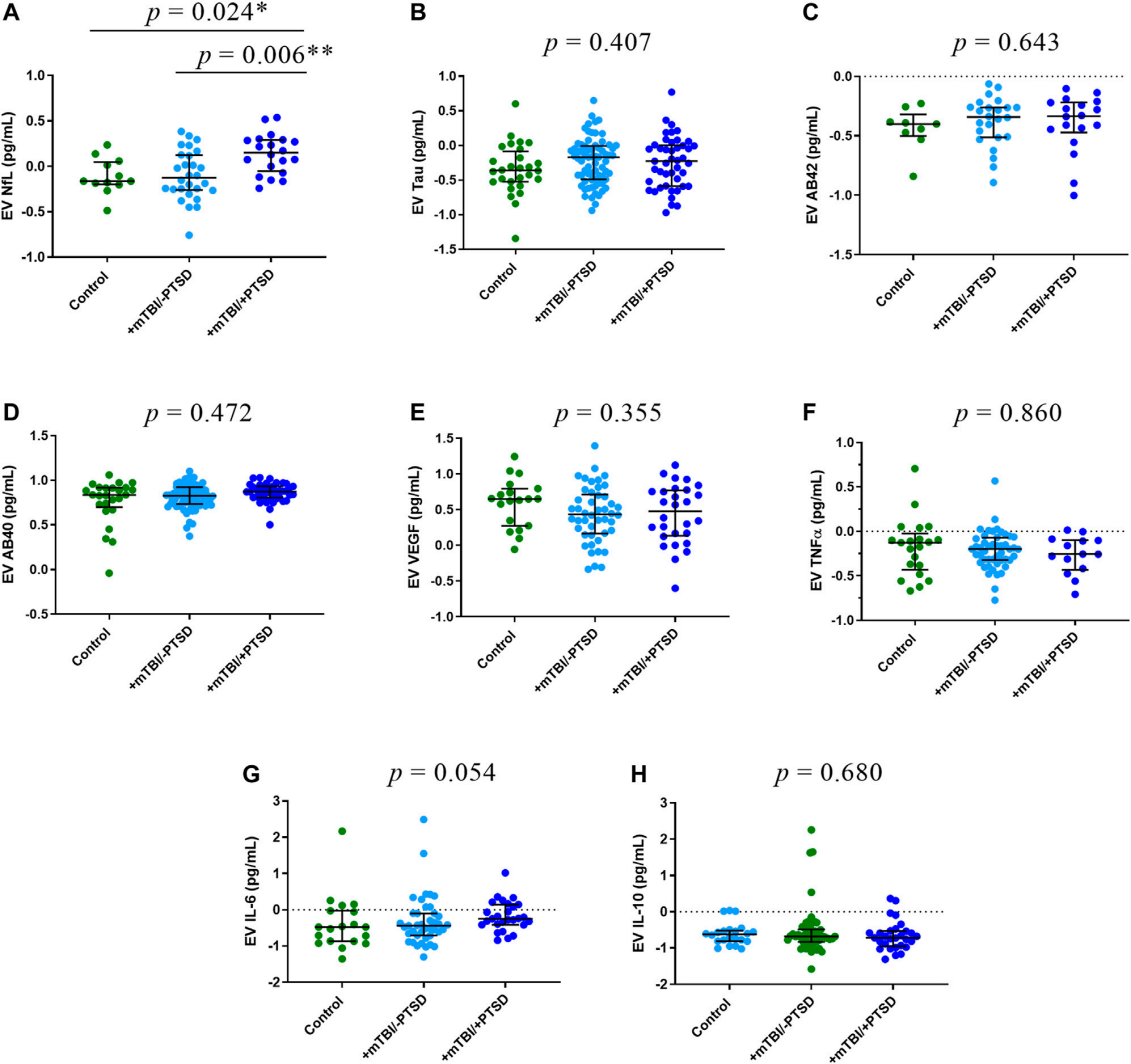
EV levels of protein biomarkers. Participants were classified into three groups based on TBI history and severity of PTSD symptoms as measured by the PTSD Checklist for DSM-5 (PCL-5): 1) Control (i.e., -TBI and -PTSD); 2) +mTBI/-PTSD; 3) +mTBI/+PTSD. Group comparisons were performed by using Kruskal-Wallis test followed by Dunn’s test, and Bonferroni method to correct for multiple comparisons. *p* values refer to pairwise comparisons **(A)** or to overall group comparisons **(B–H)**. Biomarker concentrations were represented as median ± IQR. Concentrations were log transformed to improve data visualization in the graphs. Abbreviations: Neurofilament light (NfL), Amyloid beta 42 (AB42), Amyloid beta 40 (AB40), interleukin 6 (IL-6), interleukin 10 (IL-10), tumor necrosis factor -alpha (TNFα), and endothelial growth factor (VEGF).

**FIGURE 3:**
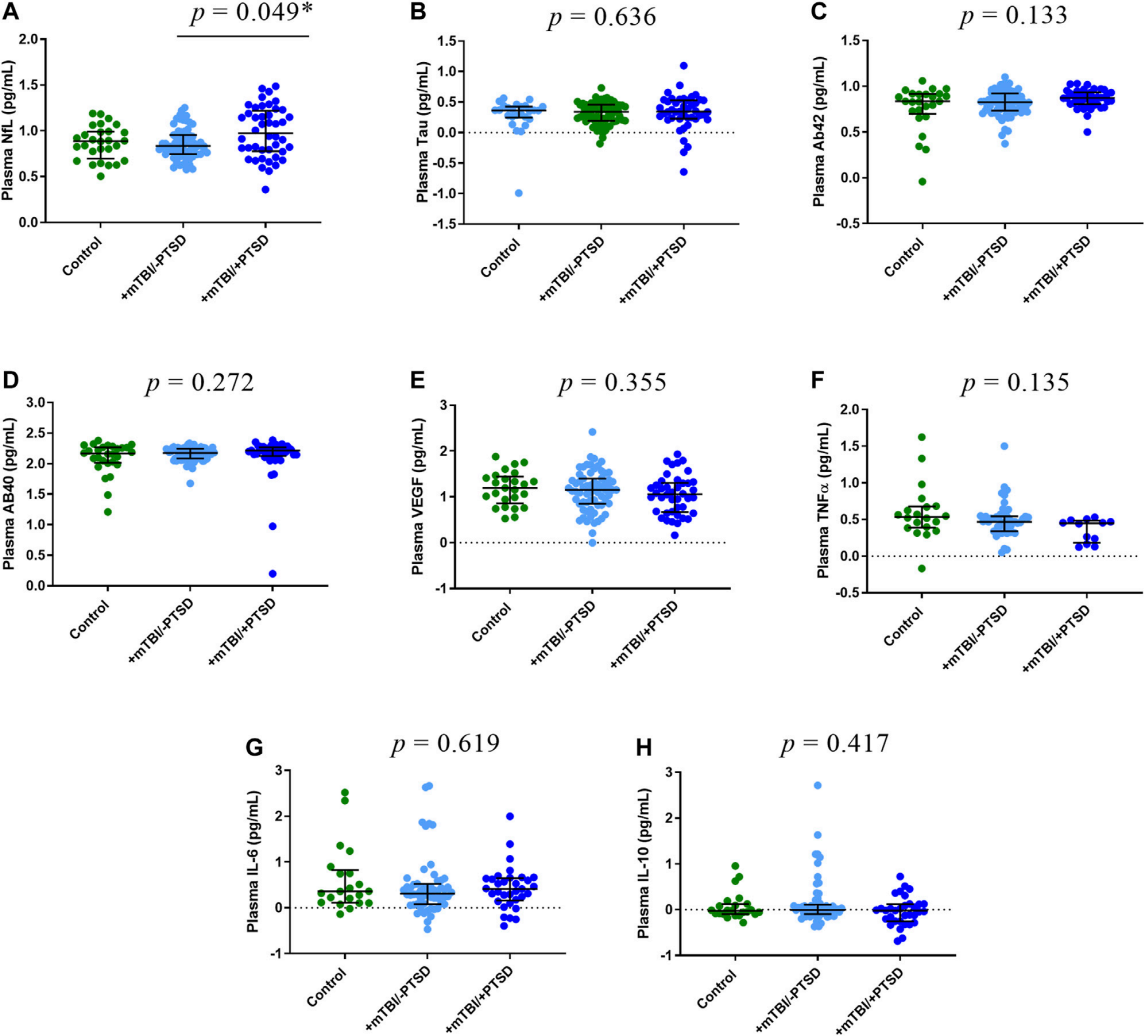
Plasma levels of protein biomarkers. Participants were classified into three groups based on TBI history and severity of PTSD symptoms as measured by the PTSD Checklist for DSM-5 (PCL-5): 1) Control (i.e., -TBI and -PTSD); 2) +mTBI/-PTSD; 3) +mTBI/+PTSD. Group comparisons were performed by using Kruskal-Wallis test followed by Dunn’s test, and Bonferroni method to correct for multiple comparisons. *p* values refer to pairwise comparisons **(A)** or to overall group comparisons **(B–H)**. Biomarker concentrations were represented as median ± IQR. Concentrations were log transformed to improve data visualization in the graphs. Abbreviations: Neurofilament light (NfL), Amyloid beta 42 (AB42), Amyloid beta 40 (AB40), interleukin 6 (IL-6), interleukin 10 (IL-10), tumor necrosis factor -alpha (TNF-α), and endothelial growth factor (VEGF).

We compared EV (Figure 2) and plasma (Figure 3) levels of proteins among control, +mTBI/-PTSD, and +mTBI/+PTSD groups (Supplementary Table S3). Significant group differences were found for EV NfL (*p* = 0.003) and plasma NfL (*p* = 0.048). Pairwise comparison showed higher levels of EV NfL in the +mTBI/+PTSD when compared to the +mTBI/-PTSD (*p* = 0.006) and control groups (*p* = 0.024). For plasma NfL, pairwise comparisons showed significant differences between + mTBI/+PTSD and +mTBI/-PTSD groups (*p* = 0.049). No other significant differences among groups were observed. Group differences for EV IL-6 levels were marginally significant (*p* = 0.054).

To evaluate associations between PTSD symptoms and levels of proteins in EV and plasma, we performed correlation analysis (Figure 1, Supplementary Table S2). EV NfL (*ρ* = 0.41, *p* = 0.004) and plasma NfL (*ρ* = 0.26, *p* = 0.006) correlated with PCL-5 scores. No other significant correlations between PCL-5 and proteins were observed. Marginally significant correlations were observed between PCL-5 scores and EV IL-6 (*ρ* = 0.22, *p* = 0.078), plasma TNFα (*ρ* = -0.19, *p* = 0.093), and EV Aβ40 (*ρ* = 0.28, *p* = 0.086). To further investigate associations between PCL-5 scores and biomarker levels, we built linear regression models including possible confounders such as total number of mTBIs and time since last TBI. We observed a significant association between PCL-5 scores levels of EV NfL and plasma NfL (Table 3). Additionally, we found a marginally significant association between plasma VEGF and PCL-5 scores (*p* = 0.073).

## Discussion

This cross-sectional study evaluated peripheral blood EV levels of miRNAs and a panel of proteins among SMs/Vs with prior combat deployment, and their associations with mTBI history and current PTSD symptoms measured by the PCL-5 questionnaire. Participants who sustained one or more mTBIs were divided into two groups according to their PCL-5 scores. The control group consisted of those negative for any lifetime TBI and having lower PTSD symptom severity scores (PCL-5 below 38). We observed elevated EV and plasma levels of NfL in SMs/Vs with mTBI and more severe PTSD symptoms (PCL-5 score of 38 or higher). Moreover, changes in expression profiles of EV miRNAs were linked to PTSD symptom burden. These findings contribute to the efforts to develop prognostic biomarkers of TBI-related behavioral health disorders, and shed light on possible molecular mechanisms associated with the development of persistent PTSD symptoms in TBI populations.

EV biological functions in health and disease are yet to be completely understood (Margolis and Sadovsky, 2019; Guedeset al., 2020b). Short and long distance cell-to-cell communication mediated by EVs has been established as one of their important biological functions (Valadi et al., 2007; Frühbeis et al., 2013; Raposo and Stoorvogel, 2013; Cocucci and Meldolesi, 2015; Fitzgerald et al., 2018). EVs might also contribute to central nervous system (CNS) protective mechanisms as they assist in clearance processes such as the removal of unwanted proteins (Bulloj et al., 2010; Dinkins et al., 2017). They may also play a role in the development of neurodegenerative conditions as EVs have been linked to packaging and spreading of misfolded proteins, a mechanism shared by a number of neurodegenerative diseases (Perez-Gonzalez et al., 2012; Budnik et al., 2016; Thompson et al., 2016; Dinkins et al., 2017). In Alzheimer’s disease (AD), EVs have been implicated in the lateral and long-distance propagation of tau and might contribute to the biogenesis of Aβ fragments (Sharples et al., 2008; Bulloj et al., 2010; Perez-Gonzalez et al., 2012). Additionally, EVs play roles in inflammatory responses and angiogenesis (Gupta and Pulliam, 2014; Todorova et al., 2017; Fitzgerald et al., 2018; Oggero et al., 2019). Our group has explored the potential of EVs as biomarkers in TBI in previous studies. We have shown higher levels of EV NfL (Guedes et al., 2020a) and EV tau (Kenney et al., 2018) in individuals with history of 3 or more mTBIs when compared to those with 1 or 2 mTBIs. In addition, we observed links between higher levels of EV NfL and EV tau and more severe neurobehavioral and PTSD symptoms (Kenney et al., 2018; Guedes et al., 2020a). Interestingly, we have also shown associations between EV levels of specific miRNAS, including hsa-miR-139–5p, and severity of neurobehavioral symptoms (Devoto et al., 2020).

In this study, levels of EV NfL were higher among participants with TBI history and more severe PTSD symptoms when compared to those with less PTSD symptoms and controls. EV NfL was also associated with symptom severity in correlation analysis and regression models controlling for demographics, number of TBIs and time since last injury. In addition, a marginally significant correlation between EV Aβ40 and PCL-5 scores was also found. A broad literature supports the relevance of NfL as a biomarker of nervous system pathologies involving axonal injury or degeneration, including mTBI (Neselius et al., 2012; Bacioglu et al., 2016; Shahim et al., 2016; Yuan et al., 2017). Higher levels of NfL in blood have been observed in several neuroinflammatory and neurodegenerative conditions such as AD (Menke et al., 2015; Meeter et al., 2016; Preische et al., 2019). Elevated NfL in blood has also been shown to correlate with severity of chronic depression, postconcussive and PTSD symptoms in mTBI patients (Guedes et al., 2020a; Shahim et al., 2020). Here, we also observed links between the severity of PTSD symptoms and the levels of miRNAs that have been previously associated with some pathologies of neurodegenerative diseases. Our findings suggest a connection between the severity of persistent chronic PTSD symptoms years after mTBI and neurodegenerative changes. PTSD has been associated with structural alterations in areas of the frontal cortex, hippocampus and amygdala in animal models and human subjects, and functional changes within neural networks (Pitman et al., 2012; Fan et al., 2019; Zandvakili et al., 2020). We hypothesized that changes in brain connectivity with chronic axonal pathology might be, at least in part, underlying links between TBI and persistent PTSD symptoms. Further studies are necessary to evaluate possible roles of neurodegenerative changes and cell signaling mechanisms involving EVs in the development of persistent PTSD symptoms following TBI.

In the miRNA analysis, hsa-miR-3196, hsa-miR-372–3p, hsa-miR-615–5p, hsa-miR-1185-1-3p, hsa-miR-3190–3p, and hsa-miR-139–5p were differentially regulated in participants with more severe TBI symptoms. We observed a downregulation of hsa-miR-374a-3p in participants with mTBI and more severe PTSD symptoms in comparison to those with mTBI and less severe symptoms. miR-374a-3p has been linked to downregulation of pro-inflammatory markers associated with insulin resistance (Doumatey et al., 2018), and targets molecules that are part of oxidative phosphorylation, mitochondrial dysfunction, neuroinflammation, and amyotrophic lateral sclerosis canonical signaling pathways, among others. In addition, we found that hsa-miR-139–5p was downregulated in the mTBI groups when compared to controls and linked to severity of PTSD symptoms in regression models. Moreover, regression models including EV or plasma NfL were improved with the inclusion of hsa-miR-139–5p. Importantly, hsa-miR-139–5p is also suppressed in the blood of rats resilient to chronic stress, suggesting a link between this miRNA and vulnerability to stress (Chen et al., 2015), but possible associations between hsa-miR-139–5p and TBI-related PTSD symptoms had not been previously reported.

Our finding of downregulated hsa-miR-139–5p in the mTBI groups also confirms previous studies in animal models, showing a downregulation of hsa-miR-139–5p in the dentate gyrus in association with chronic mTBI (Puhakka et al., 2017). Decrease in peripheral blood EV hsa-miR-139–5p in Alzheimer’s disease (AD) patients when compared to controls has also been reported (Lugli et al., 2015). hsa-miR-139–5p has been implicated in neurodegenerative processes in an AD model via the targeting of metabolism- and circadian rhythm-related genes, and is considered a candidate biomarker for prion diseases (Noh et al., 2014). hsa-miR-139–5p targets CASP3, which encodes the caspase 3 protein. CASP3 is part of canonical pathways functionally associated with mitochondrial dysfunction, oxidative phosphorylation, amyotrophic lateral sclerosis, Huntington’s disease Signaling, and Parkinson’s disease signaling, among others. Caspase 3 is important in the activation cascade of caspases in apoptosis, and a major mediator of apoptosis in neurons (Brentnall et al., 2013; D’Amelio et al., 2009). Moreover, caspase 3 is involved in the cleavage of β-amyloid precursor protein (APP), which is targeted by hsa-miR-372–3p and linked to cell death in AD (Nishimura et al., 2002). APP cleavage by caspase 3 also occurs in rodent models of traumatic axonal injury (Stone et al., 2002). Other components of the amyloid processing pathway are the calpain small subunit 1 gene (CAPNS1), a hsa-miR-3196 target, and protein kinase cAMP-activated catalytic subunit beta (PRKACB), which is also part of axonal guidance and synaptogenesis signaling pathways and targeted by hsa-miR-372–3p.

The cAMP Response Element-Binding Protein (CREB) signaling in neurons canonical pathway is modulated by hsa-miR-3196, hsa-miR-372–3p, hsa-miR-615–5p, hsa-miR-1185-1-3p, hsa-miR-3190–3p, hsa-miR-374a-3p, and hsa-miR-139–5p. CREB acts as a transcription factor binding to the cAMP response element (CRE) of the promoters of its target genes (Wang et al., 2018). In neurons, CREB signaling is associated with processes such as proliferation, differentiation, neurogenesis, and plasticity (Sakamoto et al., 2011; Wang et al., 2018). CREB is a major regulator of neurotrophins such as brain derived growth factor (BDNF), and BDNF promotes the activation of CREB through tropomyosin receptor kinase (Trk) B receptors (Yossifoff et al., 2008; Wang et al., 2018). SHC1 (SHC adaptor protein 1) is part of the CREB signaling in neurons canonical pathway and a target of hsa-miR-139–5p. SHC1 is also involved in several canonical pathways that include synaptogenesis, actin/cytoskeleton, VEGF, IL-6, estrogen receptor, and glucorticoid receptor signaling. The Shc family of adaptor proteins has multiple domains that allow the recruitment of multiple signaling molecules, and plays a major role in cell signaling mediated by integrins and growth factors (Ravichandran, 2001; Ahmed and Prigent, 2017).

In this study, all miRNAS that were differentially regulated in participants with more severe PTSD symptoms have target molecules that are part of the Glucocorticoid receptor (GR) signaling canonical pathway. GR, in addition to mineralocorticoid receptors, mediate the effects of Glucocorticoids (GCs), steroid hormones that are synthesized in the adrenal cortex and have potent anti-inflammatory effects (Yehuda 2009; Sundahl et al., 2015; Scheschowitsch et al., 2017). The hypothalamic–pituitary–adrenal (HPA) axis coordinates hormonal and inflammatory stress responses to stress. The activation of the HPA in response to acute stress culminates in GC release, which plays a role in the coordination of hormonal and behavioral stress responses (Leistner and Menke, 2020). Changes in the HPA axis are a characteristic of PTSD pathophysiology (Yehuda, 2009), and associations between PTSD and changes in GR sensitivity involving the FK506 binding protein 5 (FKBP5) gene recently emerged (Li et al., 2020). PTSD has also been associated with an increased risk of cardiovascular disease (CVD) (Edmondson et al., 2013; Scherrer et al., 2019; Ebrahimi et al., 2021), which might be due to comorbid conditions (Scherrer et al., 2019), and insulin resistance (Michopoulos et al., 2016; Blessing et al., 2017). Accordingly, in this study, miRNAs that were differentially regulated in participants with more severe PTSD symptoms were also associated with insulin secretion signaling, cardiac hypertrophy and cardiac beta-adrenergic signaling canonical pathways.

PTSD has been previously linked to low grade systemic inflammation, characterized by elevated blood levels of inflammatory markers (Maes et al., 1999; Passos et al., 2015; Speer et al., 2018). Chronic PTSD symptoms have been associated with peripheral blood elevations of IL-6 in civilians and military populations (Gill et al., 2010; Lindqvist et al., 2014; Rodney et al., 2020). Cytokines mediate cell-to-cell communication as soluble factors and in association with EVs, where they might be surface-bound or encapsulated (Fitzgerald et al., 2018). In this study, we observed higher levels of EV IL-6 in those with mTBI and more severe PTSD symptoms, but group comparisons were only marginally significant. miR-1185-1-3p modulates the mRNA levels of GSK3B, which has been linked to peripheral and central inflammatory diseases, and promotes the expression of cytokines such as TNF-α and IL-6 (Beurel 2011; Garcia-Lacarte et al., 2019). In addition, hsa-miR-3196, hsa-miR-372–3p, hsa-miR-615–5p, and hsa-miR-139–5p were associated with the IL-6 canonical signaling pathway as seen in the miRNA target analysis. Further investigation of links between EV levels of cytokines and the development of PTSD symptoms in military populations are warranted.

Limitations of this study include the relatively small sample size and number of participants with severe PTSD symptoms, though the largest to date exploring this novel biomaker, and the variability in number of mTBIs and years since last TBI, and results must be considered exploratory and hypothesis generating in this Biomarker Discovery dataset. Additionally, we evaluated PTSD symptoms using a questionnaire that allows for quantification of symptom severity, but is not a stand-alone diagnostic tool. Our cohort was predominantly white and male, which limits our ability to generalize our findings. Further, all were combat-deployed which limits our findings to combat-related TBI and PTSD symptom severity and is not generalizable to civilian populations. Levels of miRNA in peripheral blood may be influenced by other factors, such as medication use, that were not evaluated in this study. Furthermore, proteins and miRNAs analyzed here are found at low concentrations in the peripheral circulation. Even though we selected methods with high sensitivity to measure their concentrations, EV proteins and miRNAs were not detectable in some samples. The methods used in this study do not allow us to identify the tissue of origin, or to distinguish miRNA and proteins that are located inside the vesicles from those that are bound to the EV membrane. Future technical advancements could further improve EV analysis as well as detection of molecules found at low levels in the peripheral circulation that might reflect pathological processes underlying the development of PTSD symptoms following mTBIs.

Despite these limitations, our findings could inform subsequent larger prospective studies examining longitudinal changes in EV biomarkers, which are warranted to validate our results and to understand the role of EVs in the pathophysiology of chronic mTBI and PTSD. Longitudinal studies could identify relationships between levels of biomarkers and PTSD symptoms over time, and biomarkers that could predict those most at risk of developing severe PTSD symptoms at later timepoints. This line of research may lead to novel avenues for the treatment of PTSD, and may facilitate clinical interventions prior to the onset of symptoms and underlying pathological processes.

## Conclusion

Here, we found links between levels of NfL and the severity of PTSD symptoms in persons with military combat deployment and varying mTBI histories. We also observed an association between persistent PTSD symptoms and the EV levels of miRNAs, especially hsa-miR-139–5p. Our results shed light on possible mechanisms underlying individual susceptibility to the development of persistent or later-in-life PTSD symptoms. Specifically, NfL and hsa-miR-139–5p were linked to the severity of PTSD symptoms in group comparisons, correlation analysis and regression models controlling for potential confounders. NfL is a marker of axonal damage that is elevated in neurodegenerative conditions. Similarly, hsa-miR-139–5p has been previously associated to neurodegenerative processes. Our results suggest a possible role for axonal degeneration and neurodegenerative changes in the development of persistent chronic PTSD symptoms years after mTBI.

## Data Availability

The original contributions presented in the study are included in the article/Supplementary Materials, further inquiries can be directed to the corresponding author.
