# Supplementary figures and images for: Extracellular Vesicle Proteins and MicroRNAs Are Linked to Chronic Post-Traumatic Stress Disorder Symptoms in Service Members and Veterans With Mild Traumatic Brain Injury
Source: Front Pharmacol. 2021 Oct 6;12:745348. doi: 10.3389/fphar.2021.745348 (PMC8526745; doi:10.3389/fphar.2021.745348)

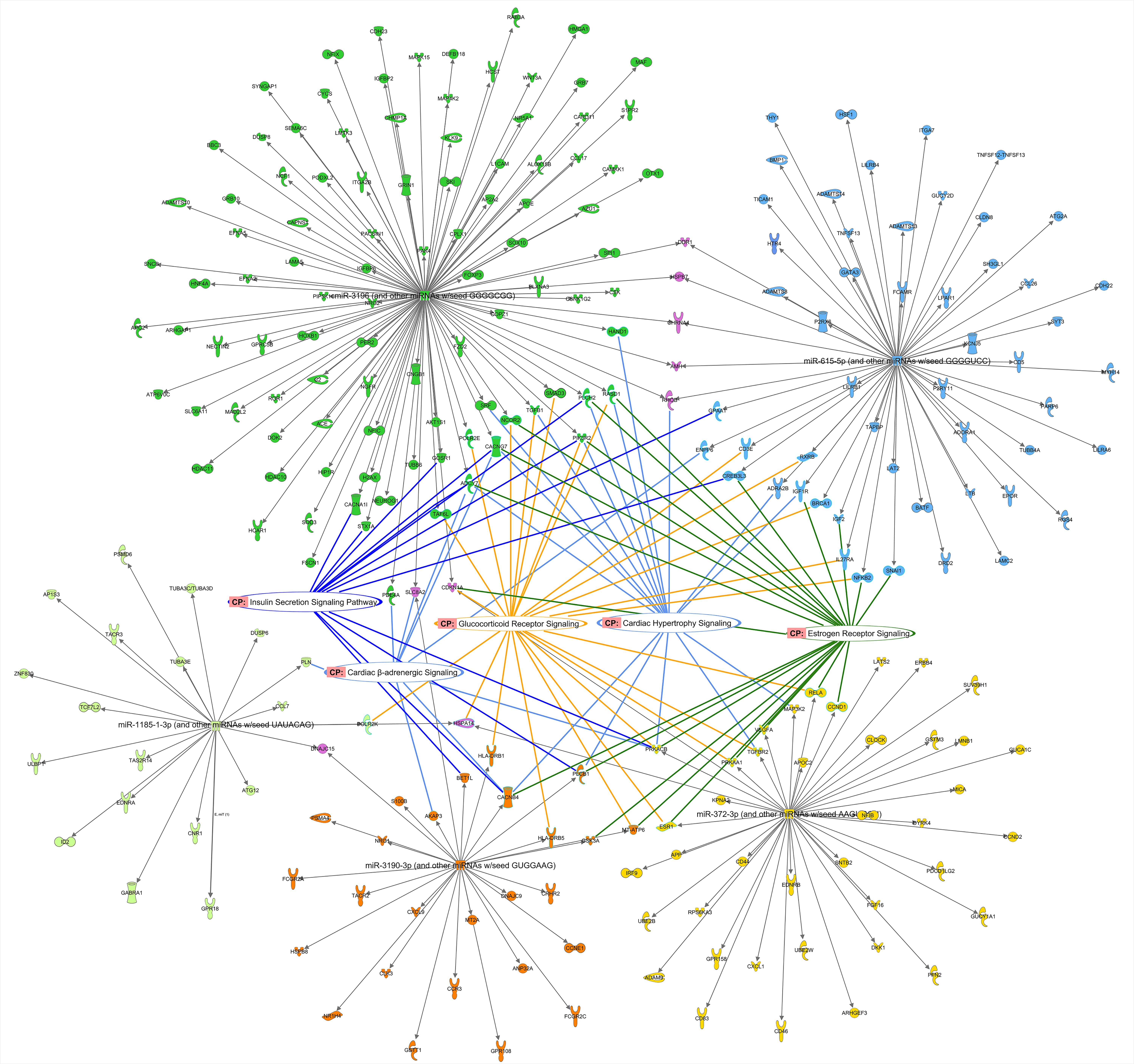

Supplement: Supplementary file 2 [file Image4.tif]

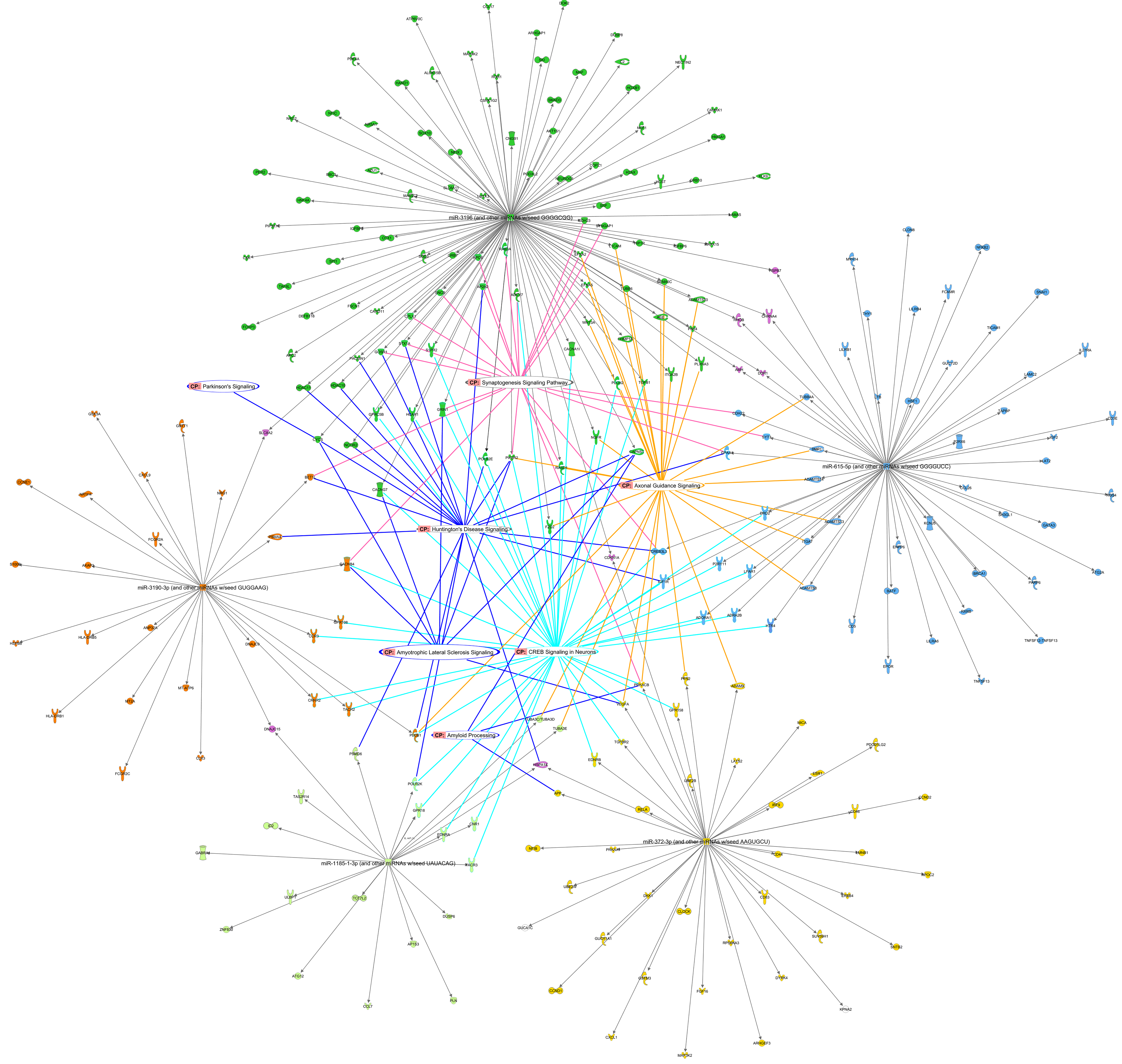

Supplement: Supplementary file 3 [file Image2.tif]

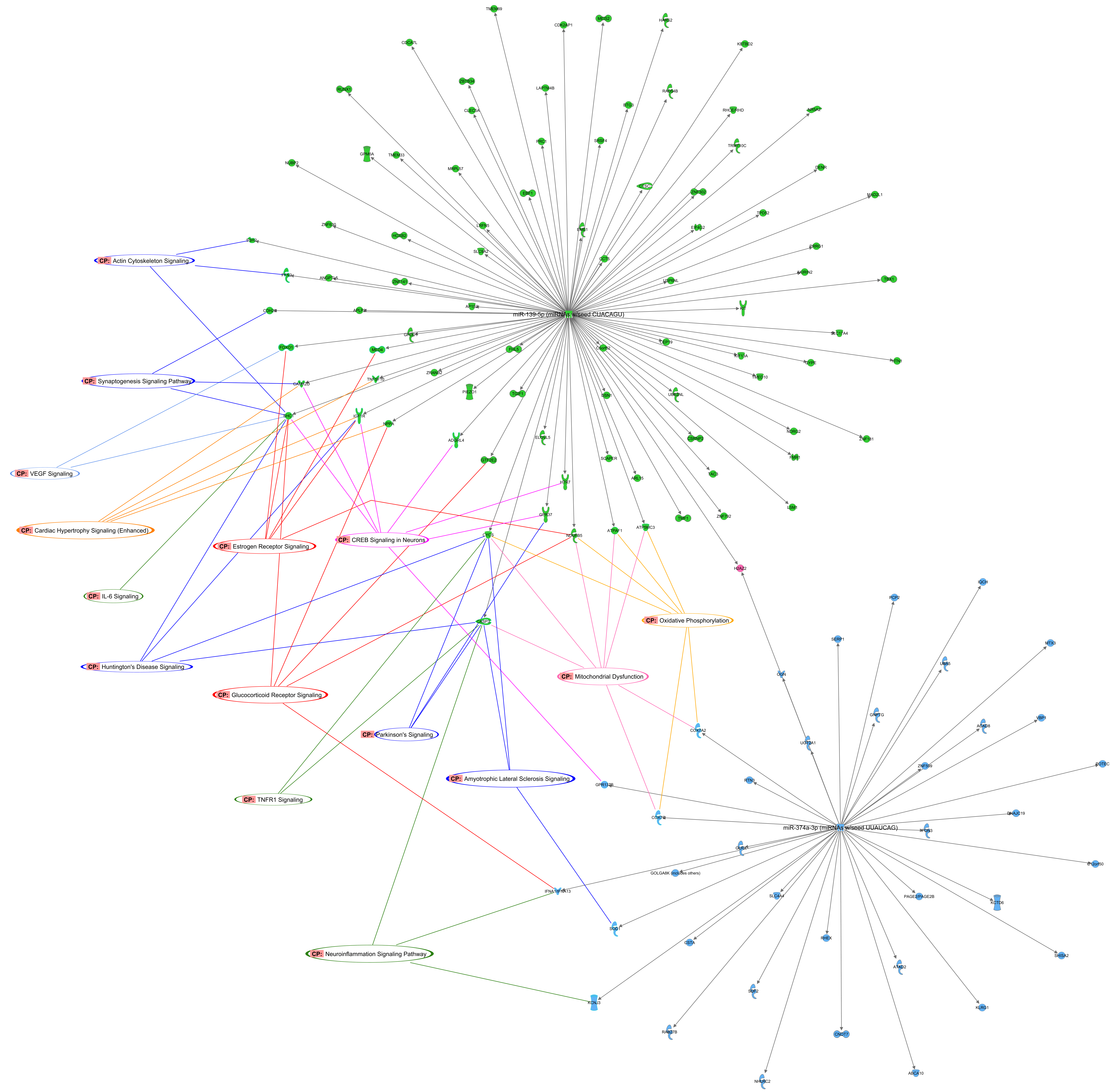

Supplement: Supplementary file 4 [file Image1.tif]
