# Supplementary material for: Extracellular Vesicle Proteins and MicroRNAs Are Linked to Chronic Post-Traumatic Stress Disorder Symptoms in Service Members and Veterans With Mild Traumatic Brain Injury
Source: Front Pharmacol. 2021 Oct 6;12:745348. doi: 10.3389/fphar.2021.745348 (PMC8526745; doi:10.3389/fphar.2021.745348)
Supplement: Supplementary file 5 [file DataSheet1.pdf]

Supplementary Material

1 Supplementary Tables

1.1 Supplementary Table 1: Average miRNA counts of differentially expressed miRNAs

Characteristic	Target Sequence[a]	Control (-TBI/-PTSD) (n=28)[1]	+mTBI/-PTSD (n=71)[1]	+mTBI/+PTSD (n=45)[1]	p-value[2]
**hsa-miR-139-5p**	UCUACAGUGCACGUGUCUCCAG	9.00 (7.75, 11.00)	6.00 (5.00, 8.00)	7.00 (5.00, 9.00)	**<0.001**
**hsa-miR-3190-3p**	UGUGGAAGGUAGACGGCCAGAGA	5.50 (4.00, 7.00)	6.00 (5.00, 8.00)	7.00 (6.00, 9.00)	**0.016**
**hsa-miR-204-5p**	UUCCCUUUGUCAUCCUAUGCCU	6.0 (4.8, 8.0)	8.0 (6.0, 11.0)	8.0 (6.0, 10.0)	**0.020**
**hsa-miR-1185-1-3p**	AUAUACAGGGGGAGACUCUUAU	11.0 (8.0, 14.0)	10.0 (9.0, 16.0)	14.0 (12.0, 18.0)	**0.027**
**hsa-miR-374a-3p**	CUUAUCAGAUUGUAUUGUAAUU	7.0 (6.0, 9.0)	9.0 (7.0, 10.0)	7.0 (5.0, 9.0)	**0.028**
**hsa-miR-615-5p**	GGGGGUCCCCGGUGCUCGGAUC	6.0 (4.0, 7.0)	8.0 (5.0, 10.0)	8.0 (5.0, 11.0)	**0.030**
**hsa-miR-372-3p**	AAAGUGCUGCGACAUUUGAGCGU	6.0 (5.0, 9.0)	9.0 (6.0, 11.0)	8.0 (7.0, 10.0)	**0.031**

Characteristic	Target Sequence[a]	Control (-TBI/-PTSD) (n=28)[1]	+mTBI/-PTSD (n=71)[1]	+mTBI/+PTSD (n=45)[1]	p-value[2]
**hsa-miR-509-3-5p**	UACUGCAGACGUGGCAAUCAUG	6.0 (5.0, 8.0)	8.0 (6.0, 10.0)	7.0 (6.0, 10.0)	**0.035**
**hsa-miR-3196**	CGGGGCGGCAGGGGCCUC	4.50 (3.00, 5.25)	6.00 (3.00, 9.00)	6.00 (4.00, 8.00)	**0.040**
**hsa-miR-1277-3p**	UACGUAGAUAUAUAUGUAUUUU	7.0 (5.8, 9.0)	10.0 (8.0, 12.5)	9.0 (6.0, 11.0)	**0.045**
**hsa-miR-425-5p**	AAUGACACGAUCACUCCCGUUGA	5.0 (4.0, 7.0)	7.0 (5.0, 9.0)	6.0 (3.0, 8.0)	**0.046**
**hsa-miR-375**	UUUGUUCGUUCGGCUCGCGUGA	5.0 (3.8, 6.0)	6.0 (5.0, 9.0)	6.0 (5.0, 8.0)	**0.049**

[1] Statistics presented: Median (IQR)
[2] Statistical tests performed: Kruskal-Wallis test

Average normalized miRNA counts for the differentially expressed miRNAs as revealed by group comparisons. Analysis of raw miRNA data was performed using the nSolver analysis software (version 4.0, NanoString technologies). P values refer to comparisons including all three groups. [a] Sequence information as available on NanoString technologies webpage.

1.2 Supplementary Table 2: Correlations between PTSD symptoms and levels of proteins and miRNAs

Proteins			miRNAs		
	p	ρ		*p*	ρ
EV NfL	**0.004**	**0.408**	hsa-miR-139-5p	**0.006**	**0.255**
Plasma NfL	**0.006**	**0.258**	hsa-miR-1185-1-3p	**0.010**	**0.238**
EV IL-6	0.078	0.215	hsa-miR- 425-5p	0.065	-0.172
Plasma IL-6	0.589	0.058	hsa-miR-3190-3p	0.086	0.160
EV TNFα	0.981	0.003	hsa-miR-204-5p	0.796	-0.024
Plasma TNFα	0.093	-0.185	hsa-miR-372-3p	0.387	0.081
EV Aβ40	0.086	0.279	hsa-miR-509-3-5p	0.518	-0.061
Plasma Aβ40	0.143	0.137	hsa-miR-615-5p	0.770	0.027
EV Aβ42	0.488	-0.110	hsa-miR-1277-3p	0.796	-0.024
Plasma Aβ42	0.183	0.129	hsa-miR-375	0.514	0.061
EV tau	0.401	0.079	hsa-miR-3196	0.548	0.056
Plasma tau	0.268	0.106	hsa-miR-374a-3p	0.493	-0.064
EV IL-10	0.851	0.021			
Plasma IL-10	0.287	-0.113			
EV VEGF	0.453	-0.089			
Plasma VEGF	0.152	-0.138			

Spearman correlation significance levels (*p*) and coefficients (ρ) between proteins levels (left), miRNA levels (right) and PTSD Checklist for DSM-5 (PCL-5 scores). Statistically significant results are marked in bold. Abbreviations: NfL (Neurofilament light), Aβ42 (Amyloid Beta 42), Aβ40 (Amyloid Beta 40), IL-6 (interleukin 6), IL-10 (interleukin 10), TNFα (tumor necrosis factor-alpha), and endothelial growth factor (VEGF).

1.3 Supplementary Table 3: EV and plasma levels of protein biomarkers

Characteristic	Control (-TBI/-PTSD) (n=28)	+mTBI/-PTSD (n=71)	+mTBI/ +PTSD (n=45)
Plasma NfL	7.7 (5.7, 9.7)	6.8 (5.6, 8.9)	9.4 (6.1, 15.8)
EV NfL	0.69 (0.65, 1.06)	0.75 (0.56, 1.32)	1.42 (0.96, 1.94)
Plasma tau	2.31 (1.81, 2.65)	2.19 (1.56, 2.85)	2.20 (1.72, 3.38)
EV tau	0.44 (0.30, 0.72)	0.68 (0.33, 0.95)	0.59 (0.26, 1.00)
Plasma Aβ40	146 (106, 184)	150 (122, 175)	163 (136, 182)
EV Aβ40	2.84 (2.01, 3.94)	3.12 (1.44, 4.01)	3.61 (2.81, 4.59)
Plasma Aβ42	6.84 (5.36, 8.18)	6.69 (5.43, 8.36)	7.47 (6.40, 8.52)
EV Aβ42	0.40 (0.34, 0.41)	0.46 (0.31, 0.55)	0.46 (0.36, 0.59)
Plasma TNFα	3.32 (2.42, 4.67)	2.95 (2.52, 3.48)	2.80 (1.87, 3.27)
EV TNFα	0.71 (0.40, 0.86)	0.63 (0.48, 0.83)	0.61 (0.40, 0.79)
Plasma IL-6	2.28 (1, 6)	2.06 (1, 3)	2.58 (2, 4)
EV IL-6	0.33 (0.14, 0.91)	0.36 (0.20, 0.79)	0.56 (0.39, 1.30)
Plasma IL-10	0.95 (0.81, 1.29)	0.98 (0.82, 1.22)	0.96 (0.56, 1.31)

Characteristic	Control (-TBI/-PTSD) (n=28)	+mTBI/-PTSD (n=71)	+mTBI/ +PTSD (n=45)
EV IL-10	0.24 (0.16, 0.29)	0.21 (0.15, 0.31)	0.19 (0.12, 0.29)
Plasma VEGF	15.7 (9, 26)	14.2 (7, 24)	11.4 (5, 20)
EV VEGF	4.45 (2.05, 5.86)	2.72 (1.51, 4.97)	3.06 (1.44, 5.73)

EV and plasma levels of protein biomarkers (Median, interquartile range [IQR]) are provided. Participants were classified into three groups based on TBI history and severity of PTSD symptoms as measured by the PTSD Checklist for DSM-5 (PCL-5): 1) Control (i.e. -TBI and -PTSD); 2) +mTBI/-PTSD; 3) +mTBI/+PTSD. Abbreviations: Neurofilament light (NfL), Amyloid beta 42 (Aβ42), Amyloid beta (Aβ40), interleukin 6 (IL-6), interleukin 10 (IL-10), tumor necrosis factor - alpha (TNFα), and endothelial growth factor (VEGF).

2 Supplementary Figures Legends

Supplementary Figure 1. Molecular targets of hsa-miR-139-5p (green) and hsa-miR-374a-3p (blue) and associated canonical pathways. Canonical Pathways: CREB (cAMP-response element binding protein) signaling in neurons (dark pink), glucocorticoid receptor pathway (red), Huntington's disease, amyotrophic lateral sclerosis, Parkinson's disease (blue); synaptogenesis signaling pathway (blue), actin cytoskeleton signaling (blue), neuroinflammation signaling pathway (green), mitochondrial dysfunction (light pink), IL-6 signaling (green), VEGF signaling (light blue), TNFR1 signaling (green), cardiac hypertrophy signaling (orange), estrogen receptor signaling (red), oxidative phosphorylation (dark yellow). Abbreviations: CP (canonical pathways), Interleukin-6 (IL-6), tumor necrosis factor-alpha receptor 1 (TNFR1), vascular endothelial growth factor (VEGF). Target filter analysis was performed using Ingenuity Pathway Analysis (IPA) software (QIAGEN Inc).

Supplementary Figure 2. Molecular targets of hsa-miR-3196 (green), hsa-miR-615-5p (blue), hsa-miR-372-3p (yellow), hsa-miR-3190-3p (orange), and hsa-miR-1185-1-3p (light green) and associated canonical pathways. Canonical Pathways: CREB (cAMP-response element binding protein) signaling in neurons (light blue), Huntington's disease (dark blue); amyotrophic lateral sclerosis (dark blue); Parkinson's disease (dark blue); amyloid processing (dark blue); synaptogenesis signaling pathway (pink), axonal guidance signaling (dark yellow). Abbreviations: CP (canonical pathways). Target filter analysis was performed using Ingenuity Pathway Analysis (IPA) software (QIAGEN Inc).

Supplementary Figure 3. Molecular targets of hsa-miR-3196 (green), hsa-miR-615-5p (blue), hsa-miR-372-3p (yellow), hsa-miR-3190-3p (orange), and hsa-miR-1185-1-3p (light green) and associated canonical pathways. Canonical Pathways: neuroinflammation signaling pathway (dark blue), IL-6 signaling (pink), IL-10 signaling (yellow), VEGF signaling (dark red), TNFR1 signaling (green), TNFR2 signaling (green). Abbreviations: CP (canonical pathways), Interleukin-6 (IL-6), tumor necrosis factor-alpha receptor 1 (TNFR1), tumor necrosis factor-alpha receptor 2 (TNFR2), vascular endothelial growth factor (VEGF). Target filter analysis was performed using Ingenuity Pathway Analysis (IPA) software (QIAGEN Inc).

Supplementary Figure 4. Molecular targets of hsa-miR-3196 (green), hsa-miR-615-5p (blue), hsa-miR-372-3p (yellow), hsa-miR-3190-3p (orange), and hsa-miR-1185-1-3p (light green) and associated canonical pathways. Canonical Pathways: glucocorticoid receptor pathway (dark yellow), cardiac hypertrophy signaling (light blue), cardiac beta-adrenergic signaling (light blue), estrogen receptor signaling (green), insulin secretion signaling pathway (dark blue). Abbreviations: CP (canonical pathways). Target filter analysis was performed using Ingenuity Pathway Analysis (IPA) software (QIAGEN Inc).
